# Lupus érythémateux systémique induit par l'isoniazide: une complication rare à craindre

**DOI:** 10.11604/pamj.2015.20.181.5470

**Published:** 2015-02-27

**Authors:** Mahbouba Jguirim, Amna Jbeli, Hajer Ben Brahim, Amira Mhenni, Monia Youssef, Mongi Touzi, Sawssen Zrour, Ismail Bejia, Naceur Bergaoui

**Affiliations:** 1 Service de Rhumatologie, CHU Fattouma-Bourguiba Monastir, Tunisie; 2 Service de Médecine Interne, CHU Fattouma-Bourguiba Monastir, Tunisie; 3 Service des Maladies Infectieuses, CHU Fattouma-Bourguiba Monastir, Tunisie; 4 Service de Dermatologie, CHU Fattouma-Bourguiba Monastir, Tunisie

**Keywords:** Lupus érythémateux systémique, lupus induit, isoniazide, anticorps anti-nucleaires, systemic lupus erythematosus, induced lupus, isoniazid, antinuclear antibodies

## Abstract

Le lupus induit est défini comme un syndrome lupique généralement cutanéo-articulaire secondaire à une exposition continue à un traitement et qui disparaît après arrêt de celle-ci. Nous rapportons deux cas de lupus induit par l'isoniazide. Il s'agissait de deux femmes âgées respectivement de 30 et 35 ans. Elles présentaient un lupus induit par l'isoniazide après un et deux mois de traitement d'une tuberculose ganglionnaire. La maladie s'est manifestée par des signes articulaires, une éruption cutanée, une leucopénie et une anémie. Les anticorps antinucléaires et les anticorps antihistone étaient présents dans le sérum des deux malades. L’évolution était favorable après arrêt de l'isoniazide et une corticothérapie per os. Les médicaments antituberculeux notamment l'isoniazide sont responsables d'effets indésirables fréquents. Le lupus induit doit être évoqué lorsqu'un patient présente un tableau clinico-biologique évocateur.

## Introduction

Le lupus induit est défini comme un syndrome lupique généralement cutanéo-articulaire secondaire à une exposition continue à un médicament et qui disparaît après arrêt de cette exposition. Les lupus induits par les médicaments représenteraient 10% des lupus [1]. Plusieurs médicaments sont en cause. Parmi les anti-tuberculeux, l'isoniazide est le plus incriminé suivi par la rifampicine. Le lupus induit par l'isoniazide est complètement régressif à l'arrêt du traitement. Le diagnostic est évoqué devant des signes cliniques de lupus, des anticorps antinucléaires positifs sans antécédents de lupus systémique associés à l'amélioration après arrêt du médicament en cause. Nous rapportons deux cas de lupus induit par l'Isoniazide en précisant les différentes caractéristiques cliniques, biologiques, évolutives et thérapeutiques de cette entité clinique.

## Patient et observation

### Observation 1

Mme A. R âgée de 35 ans, sans antécédents pathologiques, notamment pas de lupus, était traitée par rifampicine, isoniazide, pyrazinamide et éthambutol pour une tuberculeuse ganglionnaire pendant 2 mois, puis par bithérapie associant isoniazide et rifampicine. Après 3 mois de traitement, elle avait présenté des polyarthralgies, une éruption maculopapuleuse prurigineuse au niveau des régions photo-exposées et des plaques d'alopécie diffuses au niveau du cuir chevelu. La biologie avait noté une leucopénie à 3000/mm^3^ et une anémie à 10 g/dl alors que le reste du bilan était normal. Le bilan immunologique avait montré la présence d'anticorps antinucléaires (AAN) à un taux de 1/3200 de type moucheté avec des anticorps antihistone positifs. Les anticorps anti-ADN, anti ssa et anti-ssb étaient négatifs. Le diagnostic de lupus induit par l'isoniazide était retenu indiquant son arrêt et son remplacement par l'association éthambutol et pyrazinamide en plus de la rifampicine. La patiente était traitée par prednisolone par voie orale à la dose de 10 mg/jour et par antipaludéens de synthèse pendant six mois. L’évolution après un mois de l'arrêt de l'isoniazide était marquée par la régression complète des signes cutanés, articulaires et des anomalies biologiques. En effet, le taux des AAN recontrôlé à trois semaines d'arrêt avait diminué à 1/800 jusqu’à la négativation à six mois de même les anticorps antihistone.

### Observation 2

Mme H. M, âgée de 30 ans, était traitée aussi pour une tuberculose ganglionnaire par une quadrithérapie associant isoniazide, pyrazinamide, rifampicine et streptomycine. Après un mois de traitement, elle avait présenté une ténosynovite du troisième doigt de la main droite avec une arthrite de la cheville gauche. A la biologie, il existait une leucopénie à 3500/mm^3^, une anémie normochrome normocytaire à 10 g/dl avec un syndrome inflammatoire biologique. Les AAN étaient positifs à 1/100 de type homogène avec des anticorps anti-DNA natifs positifs à un taux significatif de 393 UI/ml. Les anticorps antihistone étaient négatifs. La conduite était d'arrêter l'isoniazide en associant une corticothérapie par prednisolone, par voie orale à la dose de 10 mg/jour, antipaludéens de synthèse et anti-inflammatoire non stéroïdien avec une bonne évolution clinique et biologique au bout de 3 mois. Une infiltration locale de la cheville par des corticoïdes était faite. Le bilan immunologique s’était négativé après 8 mois d’évolution.

## Discussion

Pour retenir le diagnostic de lupus induit, cinq critères sont nécessaires: les signes cliniques et biologiques doivent être absents avant l'administration du médicament; il doit exister la notion de prise d'un médicament connu potentiellement inducteur; les symptômes doivent être réversibles à l'arrêt de la molécule en cause dans des délais variables allant de quelques semaines à deux ans; il faut documenter la présence d'AAN avec au moins un symptôme clinique de lupus systémique; le cinquième critère est celui de la réapparition des manifestations pathologiques si jamais le médicament est réintroduit [2, 3]. L'isoniazide est classé parmi les antituberculeux majeurs de première ligne. Le lupus induit par l'isoniazide survient dans 1% des cas [4]. Il est déclenché à partir d'une dose de 300 mg/j [5]. Les premières manifestations du lupus induit apparaissent six mois à deux ans de traitement. Jean Sibilia insiste sur un délai d'au moins un mois [2, 3]. L'imputabilité de l'isoniazide dans les manifestations présentées par nos deux patientes laisse peu de place au doute. Les symptômes cliniques sont peu sévères dominés par l'atteinte articulaire et musculaire dans 50 à 90% des cas. Les signes cutanés sont présents dans 25 à 53% des cas avec des atteintes viscérales, neurologiques centrales et rénales, rares [6–9]. L'anémie, la leucopénie et la thrombopénie sont rarement observées. Le taux des AAN est classiquement peu élevé et la présence d'anticorps antihistone de type Ig G anti (H2A-H2B) est évocatrice mais non spécifique [10]. Ces derniers sont retrouvés chez plus de 80% des patients avec lupus induit. Les anticorps anti-Sm, anti-SSa, anti-SSb sont habituellement absents [2, 10]. Vingt-deux pour cent des malades traités par isoniazide développent, en moyenne après six mois, des AAN sans développer des signes cliniques de lupus ce qui justifie une surveillance clinique et biologique rapprochée mais n'impose pas l'arrêt du médicament [7, 10]. La plupart des malades s'améliorent après quelques semaines d'arrêt du traitement en cause. Lorsque les symptômes sont sévères, une corticothérapie brève de 2 à 10 semaines est indiquée. Les antipaludéens de synthèse n'ont aucune indication dans le lupus induit sauf si persistance des signes cliniques. Les symptômes durent rarement plus que six mois et les AAN peuvent persister pendant plusieurs années [7, 8].

## Conclusion

Les médicaments antituberculeux sont responsables d'effets indésirables fréquents, souvent mineurs mais parfois sévères. Le lupus induit à l'isoniazide doit être évoqué lorsqu'un patient présente un tableau clinico-biologique évocateur de lupus.
